# Using machine learning methods to predict 28-day mortality in patients with hepatic encephalopathy

**DOI:** 10.1186/s12876-023-02753-z

**Published:** 2023-04-06

**Authors:** Zhe Zhang, Jian Wang, Wei Han, Li Zhao

**Affiliations:** 1 Department of Gastroenterology, Tangdu Hospital, Fourth Military Medical University, No. 1 Xinsi Road, Xi’an, 710038 China; 2 Department of Neurosurgery, Tangdu Hospital, Fourth Military Medical University, No. 1 Xinsi Road, Xi’an, China

**Keywords:** Hepatic encephalopathy, 28-day mortality, Machine learning, Model interpretation

## Abstract

**Background:**

Hepatic encephalopathy (HE) is associated with marked increases in morbidity and mortality for cirrhosis patients. This study aimed to develop and validate machine learning (ML) models to predict 28-day mortality for patients with HE.

**Methods:**

A retrospective cohort study was conducted in the Medical Information Mart for Intensive Care (MIMIC)-IV database. Patients from MIMIC-IV were randomized into training and validation cohorts in a ratio of 7:3. Training cohort was used for establishing the model while validation cohort was used for validation. The outcome was defined as 28-day mortality. Predictors were identified by recursive feature elimination (RFE) within 24 h of intensive care unit (ICU) admission. The area under the curve (AUC) and calibration curve were used to determine the predictive performance of different ML models.

**Results:**

In the MIMIC-IV database, 601 patients were eventually diagnosed with HE. Of these, 112 (18.64%) experienced death within 28 days. Acute physiology score III (APSIII), sepsis related organ failure assessment (SOFA), international normalized ratio (INR), total bilirubin (TBIL), albumin, blood urea nitrogen (BUN), acute kidney injury (AKI) and mechanical ventilation were identified as independent risk factors. Validation set indicated that the artificial neural network (NNET) model had the highest AUC of 0.837 (95% CI:0.774–0.901). Furthermore, in the calibration curve, the NNET model was also well-calibrated (*P* = 0.323), which means that it can better predict the 28-day mortality in HE patients. Additionally, the performance of the NNET is superior to existing scores, including Model for End-Stage Liver Disease (MELD) and Model for End-Stage Liver Disease-Sodium (MELD-Na).

**Conclusions:**

In this study, the NNET model demonstrated better discrimination in predicting 28-day mortality as compared to other models. This developed model could potentially improve the early detection of HE with high mortality, subsequently improving clinical outcomes in these patients with HE, but further external prospective validation is still required.

**Supplementary Information:**

The online version contains supplementary material available at 10.1186/s12876-023-02753-z.

## Introduction

Hepatic encephalopathy (HE), one of the most common complications of liver cirrhosis, is defined as a brain dysfunction [1]. Significant HE occurs in approximately 30–40% of patients with cirrhosis due to hepatocellular dysfunction and portosystemic shunt [2]. Typical clinical manifestations of HE include various types of neuropsychiatric disorders, such as progressive disorientation, sleep disorders, inappropriate behavior, somnolence, coma, asterixis, hypertonia, hyperreflexia, and extrapyramidal dysfunction [3, 4]. It is worth noting that HE is a serious complication of cirrhosis associated with significant mortality and heavy financial burdens [5]. To be specific, the costs associated with HE attain 11.6 billion$, and outweigh other decompensating events in liver cirrhosis [6]. Though HE patients have improved outcomes over the past decade, several studies have indicated that the prognosis and quality of life still remain poor [7]. Specifically, HE typically heralds hepatic decompensation, and its development is usually associated with high morbidity, implying the need for liver transplantation [8–10]. Accordingly, it is critical to determine an easily accessible and simple model to estimate the risk of 28-day mortality in patients with HE.

Many existing scoring systems have been used to evaluate the prognosis of liver cirrhosis patients, but none of them are targeted at HE patients. Models for End-Stage Liver Disease (MELD) scores have been widely used as predictive tools for liver disease severity [11]. Originating from the MELD algorithm, Biggins et al. proposed a new score, the Model for End-Stage Liver Disease-Sodium (MELD-Na) model, which has a more accurate predictive ability than MELD [12].

Machine learning (ML), as part of artificial intelligence, was not limited by the state of data distribution and can handle complex relationships as well as high-dimensional data [13–15]. Consequently, the study aimed to 1) identify the significant prognostic factors for HE patients from a large database, and then to construct and validate a model that predicts 28-day mortality, 2) to compare prognostic performance of this model with those of the MELD and MELD-Na scores.

## Methods

### Data source

Data of this retrospective cohort study were obtained from one sizeable critical care database: the Medical Information Mart for Intensive Care (MIMIC)-IV version 2.0 [16]. As a large, single-center, freely available database, the MIMIC-IV database, has comprehensive, high-quality data of patients admitted to the intensive care units (ICUs) at the Beth Israel Deaconess Medical Center in Boston, Massachusetts, between 2008 and 2019. This study was approved by the Institutional Review Boards of Beth Israel Deaconess Medical Center (Boston, MA, USA) and Massachusetts Institute of Technology (Cambridge, MA). Individuals who have passed the collaborative institutional training initiative examination can have an access to these databases. We completed the online course and obtained access to the database (certification number: 48120484). The study was reported according to the REporting of studies Conducted using Observational Routinely Collected Health Data (RECORD) statement [17].

### Participant selection

In this paper, we used the International Classification of Diseases (ICD)-9 code “5722” to identify the disease “Hepatic encephalopathy”. Indeed, all the patients had underlying liver cirrhosis that led to hepatic encephalopathy. We also tried to use ICD-9 code to identify the causes of HE. Initially, a total of 1940 HE patients were extracted from databases in this retrospective study. The exclusion criteria were (1) multiple ICU admissions, (2) age < 18 years (3) ICU stay < 24 h. Since all protected health information was de-identified, the requirement for individual patient consent was waived.

### Predictors of HE

Candidate predictors extracted from MIMIC-IV included baseline information and laboratory parameters. The baseline characteristics included: age, sex, race, body mass index (BMI), myocardial infarction, congestive heart failure, peripheral vascular disease, cerebrovascular disease, dementia, chronic pulmonary disease, rheumatic disease, peptic ulcer disease, diabetes, paraplegia, renal disease, malignant cancer, severe liver disease, metastatic solid tumor, acquired immunodeficiency syndrome (AIDS), temperature, mean artery pressure (MAP), heart rate, respiratory rate, red blood cell (RBC), white blood cell (WBC), hemoglobin (HGB), platelet (PLT), red cell distribution width (RDW), hematocrit (HCT), activated partial thromboplastin time (APTT), prothrombin time (PT), international normalized ratio (INR), bicarbonate, lactate, base excess (BE), anion gap, chloride, calcium, sodium, potassium, glucose, creatinine, blood urea nitrogen (BUN), total bilirubin (TBIL), albumin, alanine transaminase (ALT), aspartate transaminase (AST), alkaline phosphatase (ALP), urine output, sepsis related organ failure assessment (SOFA) and acute physiology score III (APSIII). All data used for prediction were from < 24 h after ICU admission.

### Statistical analysis

Missing data are unavoidable in the MIMIC database, and this study used multiple imputation to account for missing data. The specific missing number (%) for included variables in the dataset before imputation is shown in Supplementary Table 1.

Values are presented in Table 1 as means with standard deviations (if normal) or medians with interquartile ranges (IQR) (if non-normal) for continuous variables and total numbers with percentages for categorical variables. Proportions were compared using the χ^2^ test or Fisher’s exact test, whereas continuous variables were compared by the t-test or Wilcoxon rank sum test, if appropriate.

**Table 1:** Baseline characteristics of the MIMIC-IV cohorts

Variables	MIMIC-IV		
	Survival (*n* = 489)	Death (*n* = 112)	*P* Value
**Demographics**			
**Age (y), median (Q1, Q3)**	59.00 (51.00,67.00)	58.00 (52.00,68.00)	0.771
**Male, n (%)**	291 (59.51)	78 (69.64)	0.060
**Race, n (%)**			0.683
Black	39 (7.98)	6 (5.36)	
White	342 (69.94)	81 (72.32)	
Hispanic	26 (5.32)	4 (3.57)	
Asian	5 (1.02)	0 (0.00)	
Others	77 (15.75)	21 (18.75)	
**BMI (kg/m**^**2**^**), median (Q1, Q3)**	28.50 (24.40,32.50)	30.50 (27.15,36.00)	0.003
**Causes**			
Virus hepatitis	6 (1.23%)	0 (0.00%)	0.599
Alcoholic liver disease	247 (50.51%)	67 (59.82%)	0.094
Autoimmune hepatitis	19 (3.89%)	4 (3.57%)	1
**Coexisting disorders, n (%)**			
Myocardial infarction	16 (3.27)	4 (3.57)	0.776
Congestive heart failure	63 (12.88)	12 (10.71)	0.640
Peripheral vascular disease	23 (4.70)	4 (3.57)	0.788
Cerebrovascular disease	23 (4.70)	4 (3.57)	0.788
Dementia	0 (0.00)	1 (0.89)	0.186
Chronic pulmonary disease	100 (20.45)	14 (12.50)	0.072
Rheumatic disease	12 (2.45)	0 (0.00)	0.136
Peptic ulcer disease	40 (8.18)	1 (0.89)	0.011
Diabetes	153 (31.29)	30 (26.79)	0.412
Paraplegia	9 (1.84)	1 (0.89)	0.697
Renal disease	91 (18.61)	21 (18.75)	1.000
Malignant cancer	58 (11.86)	16 (14.29)	0.586
Metastatic solid tumor	21 (4.29)	11 (9.82)	0.034
AIDS	3 (0.61)	0 (0.00)	1.000
AKI	272 (55.62%)	91 (81.25%)	< 0.001
Ascites	230 (47.03%)	64 (57.14%)	0.068
**Vital signs (1st 24 h)**			
Temperature (°C), median (Q1, Q3)	36.80 (36.50,37.00)	36.60 (36.40,36.90)	0.007
MAP (mmHg), median (Q1, Q3)	75.00 (68.00,83.00)	71.00 (65.00,76.50)	< 0.001
Heart rate (/min), median (Q1, Q3)	87.00 (75.00,98.00)	95.00 (82.00,108.00)	< 0.001
Respiratory rate (/min), median (Q1, Q3)	18.00 (16.00,21.00)	19.00 (17.00,24.00)	< 0.001
**Laboratory findings (1st 24 h)**			
RBC (10^9^/L), median (Q1, Q3)	3.00 (2.70,3.50)	2.95 (2.50,3.50)	0.133
WBC (× 10^9^/L), median (Q1, Q3)	8.55 (5.80,12.33)	11.40 (7.35,15.95)	< 0.001
HGB (g/dl), median (Q1, Q3)	10.00 (9.00,11.00)	10.00 (9.00,12.00)	0.603
PLT (× 10^9^/L), median (Q1, Q3)	105.00 (69.00,163.00)	100.00 (63.00,134.25)	0.132
RDW (%), median (Q1, Q3)	17.10 (15.40,18.90)	17.45 (15.78,19.65)	0.041
HCT (%), median (Q1, Q3)	29.00 (26.00,33.00)	29.00 (25.00,33.25)	0.686
APTT (seconds), median (Q1, Q3)	39.30 (33.20,49.80)	45.95 (38.10,57.77)	< 0.001
PT (s), median (Q1, Q3)	18.50 (15.70,22.50)	24.30 (19.55,30.25)	< 0.001
INR, median (Q1, Q3)	1.70 (1.40,2.10)	2.30 (1.80,2.80)	< 0.001
Bicarbonate (mmol/L), median (Q1, Q3)	23.00 (19.00,25.00)	21.00 (17.00,24.00)	0.001
Lactate (mmol/L), median (Q1, Q3)	3.00 (1.90,5.20)	4.10 (3.00,8.00)	< 0.001
BE (mEq/L), median (Q1, Q3)	-1.50 (-5.12;0.59)	-2.94 (-6.00;0.00)	0.073
Anion gap, median (Q1, Q3)	14.50 (12.45,17.22)	17.30 (14.65,22.00)	< 0.001
Chloride (mmol/L), median (Q1, Q3)	104.00 (99.00,108.00)	102.00 (96.00,107.00)	0.002
Calcium (mmol/L), median (Q1, Q3)	9.00 (8.00,9.00)	9.00 (8.00,9.00)	0.221
Sodium, (mmol/L), median (Q1, Q3)	137.00 (134.00,141.00)	136.00 (131.75,141.00)	0.163
Potassium (mmol/L), median (Q1, Q3)	5.00 (4.00,5.00)	5.00 (4.00,5.00)	0.614
Glucose (mmol/L), median (Q1, Q3)	124.00 (103.75,158.00)	116.00 (95.00,152.00)	0.043
Creatinine, mg/dL, median (Q1, Q3)	1.20 (0.80,2.00)	1.85 (1.20,3.73)	< 0.001
BUN, mg/dL, median (Q1, Q3)	28.50 (16.00,49.00)	42.35 (25.75,63.47)	< 0.001
TBIL (μmol/L), median (Q1, Q3)	3.90 (1.80,8.50)	9.15 (4.20,21.98)	< 0.001
Albumin (mmol/L), median (Q1, Q3)	3.00 (2.50,3.40)	3.00 (2.40,3.30)	0.394
ALT (U/L) (median (Q1, Q3)	38.00 (23.00,93.25)	47.50 (30.75,75.00)	0.162
AST (U/L) (median (Q1, Q3)	74.00 (44.00,178.75)	103.50 (54.00,198.00)	0.091
ALP (U/L) (median (Q1, Q3)	102.00 (72.00,157.50)	110.65 (81.75,167.25)	0.120
Urine output (ml), median (Q1, Q3)	1183.00 (701.00,1960.00)	735.00 (347.25,1403.50)	< 0.001
**Therapy strategy (1st 24 h), n (%)**			
Vasopressor, n (%)	115 (23.52)	42 (37.50)	0.004
Ventilation, n (%)	350 (71.57)	90 (80.36)	0.076
**Scoring system**			
SOFA	8.00 (6.00,11.00)	12.00 (9.00,15.00)	< 0.001
MELD	26.00 (18.00,32.00)	33.00 (28.00,40.00)	< 0.001
MELD-Na	26.00 (16.00,33.00)	35.00 (26.75,40.00)	< 0.001
APSIII	60.00 (46.00,78.00)	91.00 (69.00,110.25)	< 0.001

*MIMIC-IV* Medical Information Mart for Intensive Care-IV, *BMI* Body mass index, *AIDS* Acquired immunodeficiency syndrome, *MAP* Mean artery pressure, *RBC* Red blood cell, *WBC* White blood cell, *HGB* Hemoglobin, *PLT* Platelet, *RDW* red cell distribution width, *HCT* Hematocrit, *APTT* Activated partial thromboplastin time, *PT* Prothrombin time, *INR* International normalized ratio, *BE* Base excess, *BUN* Blood urea nitrogen, *TBIL* Total bilirubin, *ALT* Alanine transaminase, *AST* Aspartate transaminase, *ALP* Alkaline phosphatase, *SOFA* Sepsis related organ failure assessment, *MELD* Model for End-Stage Liver Disease, *MELD-Na* the Sodium for End-Stage Liver Disease, *APSIII* Acute physiology score III

**Fig. 1:**
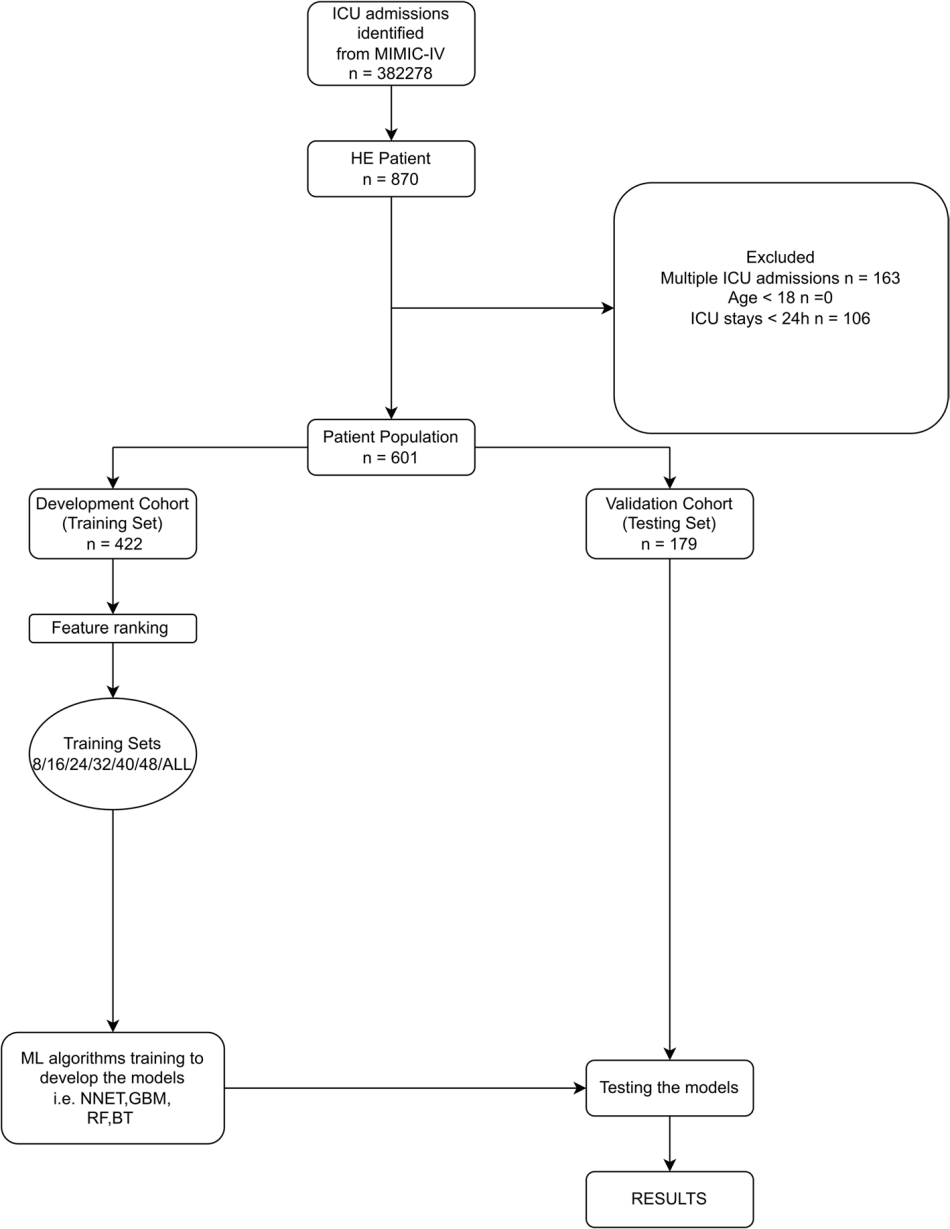
Study flow diagram and methods used for data extraction, training, and testing. ICU, intensive care unit; MIMIC-IV, Medical Information Mart for Intensive Care-IV; HE, hepatic encephalopathy; ML, machine learning; NNET, artificial neural network; GBM, gradient boosting machine; RF, random forest; BT, bagged trees

Recursive feature elimination (RFE) was used as a feature selection method in this study. Specifically, RFE in this paper is based on random forest (RF). In brief, the RFE always fits the model in according with smaller sets of features until it reaches a specified termination criterion. Then, in every cycle of the trained model, the features are ranked by importance. Finally, dependency and collinearity are eliminated. Features were considered in groups of 8/16/24/32/40/48/ALL (ALL = 51 variables, Fig. 1), according to the ranks obtained after the feature selection method.

Then, four different ML algorithms were used to develop models, including artificial neural networks (NNET), gradient boosting machine (GBM), RF, and bagged trees (BT). Initially, we randomly assigned 70% of patients in MIMIC-IV database to the training cohort and 30% to the validation cohort. The training cohort was used to establish the model, while the validation cohort was used to perform validation. When constructing the model, we employed internal validation to evaluate the stability of the prediction model in the development sets. We used ten-fold cross-validation as the resampling method to find the optimal hyperparameters; nine folds were used for training in each iteration, and the last fold was processed to tune the hyperparameters. This process was repeated 30 times. In this way, each sample was involved in both the training and testing models so that all data were used to the maximum. Next, validation was employed to evaluate the validity of each model in the validation set.

After this, all models were assessed using multiple metrics based on the model performance. We calculated the median and 95% confidence intervals (CIs) of the area under the curve (AUC), accuracy, sensitivity, specificity, negative predictive value, and positive predictive value as measures of model performance.

We used the R packages "iml" and “Shapley values” to evaluate the importance of the variables included in the model. The Shapley values can be used to enhance ML’s interpretability to describe the relative contribution of each variable within each predictive model. Specifically, The Shapley values evaluate the importance of included feature A for variables produced by all feature combinations (rather than A).

All analyses were performed using the statistical software package R version 4.0.2 (http://www.R-project.org, The R Foundation). *P*-values < 0.05 (two-sided test) were considered statistically significant.

## Results

### Baseline characteristics

As shown in Fig. 1, there were 870 patients with HE in the MIMIC-IV database; of these, 601 were eligible for this study after exclusion. In this study, 489 (81.34%) patients still survived whereas 112 (18.64%) patients died within 28 days. The process of data extraction, training preparation, and data testing using different ML algorithms was also indicated in the Fig. 1. Individuals who died were more likely to have worse baseline conditions than survivors. Causes of HE mainly included virus hepatitis, alcoholic liver disease, autoimmune hepatitis. Details are listed in Table 1.

### Variable importance

The RFE algorithm selected the following 8 important predictors: APSIII, SOFA, INR, TBIL, albumin, BUN, AKI and mechanical ventilation. All 8 variables were used in subsequent analyses for all models in both the training and testing sets.

### Prediction performance in testing set

**Fig. 2:**
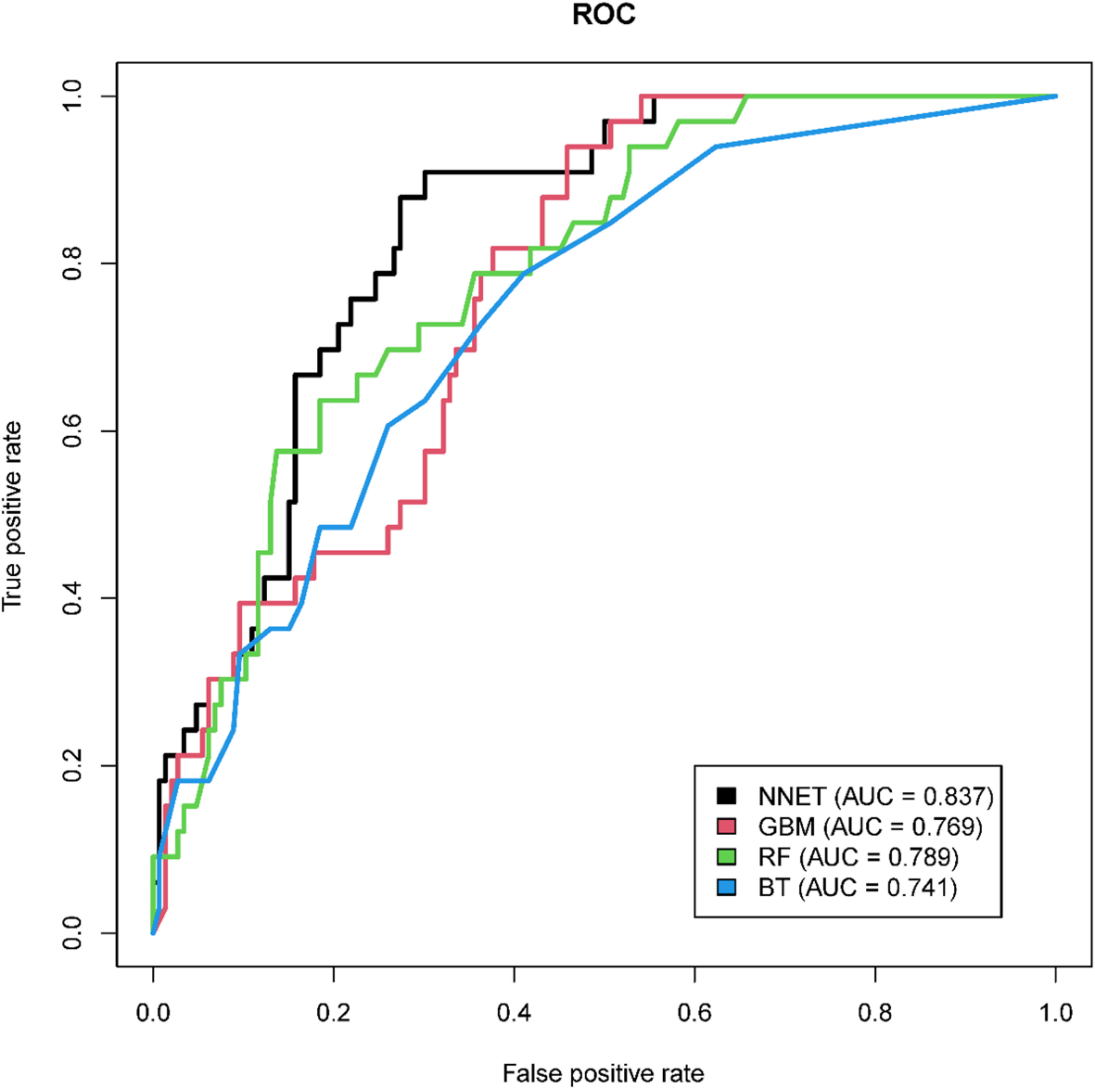
The receiver operating characteristic curves of four different machine learning models in the validation cohort. ROC, receiver operating characteristic, AUC, area under the curve; NNET, artificial neural network; GBM, gradient boosting machine; RF, random forest; BT, bagged trees

The discriminatory capabilities of all the models for predicting 28-day mortality are shown in Fig. 2 and Table 2. In the training set, NNET, GBM, RF and BT models were developed, and the testing models attained AUCs of 0.837, 0.769, 0.789, and 0.741, respectively (Fig. 2). NNET had the highest predictive performance among the four models (AUC: 0.837, 95% CI: 0.774–0.901), while the poorest discriminative ability was found in BT (AUC: 0.741, 95% CI: 0.654–0.829) (Table 2).

**Table 2:** Prediction performance of the machine learning models in the test set

Model	Sensitivity	Specificity	PPV	NPV	AUC	95% CI
NNET	0.909	0.699	0.405	0.971	0.837	(0.774,0.901)
GBM	0.939	0.541	0.316	0.975	0.769	(0.694,0.844)
RF	0.636	0.815	0.438	0.908	0.789	(0.712,0.866)
BT	0.788	0.589	0.302	0.925	0.741	(0.654,0.829)
MELD	0.768	0.579	0.295	0.916	0.728	(0.677,0.779)
MELD-Na	0.607	0.724	0.335	0.889	0.711	(0.658,0.765)

*PPV* Positive predictive values, *NPV* Negative predictive values, *AUC* Area under the curve, *CI* Confidence interval, *NNET* Artificial neural network, *GBM* Gradient boosting machine, *RF* Random forest, *BT* bagged trees, *MELD* Model for End-Stage Liver Disease, *MELD-Na* the Sodium for End-Stage Liver Disease

**Fig. 3:**
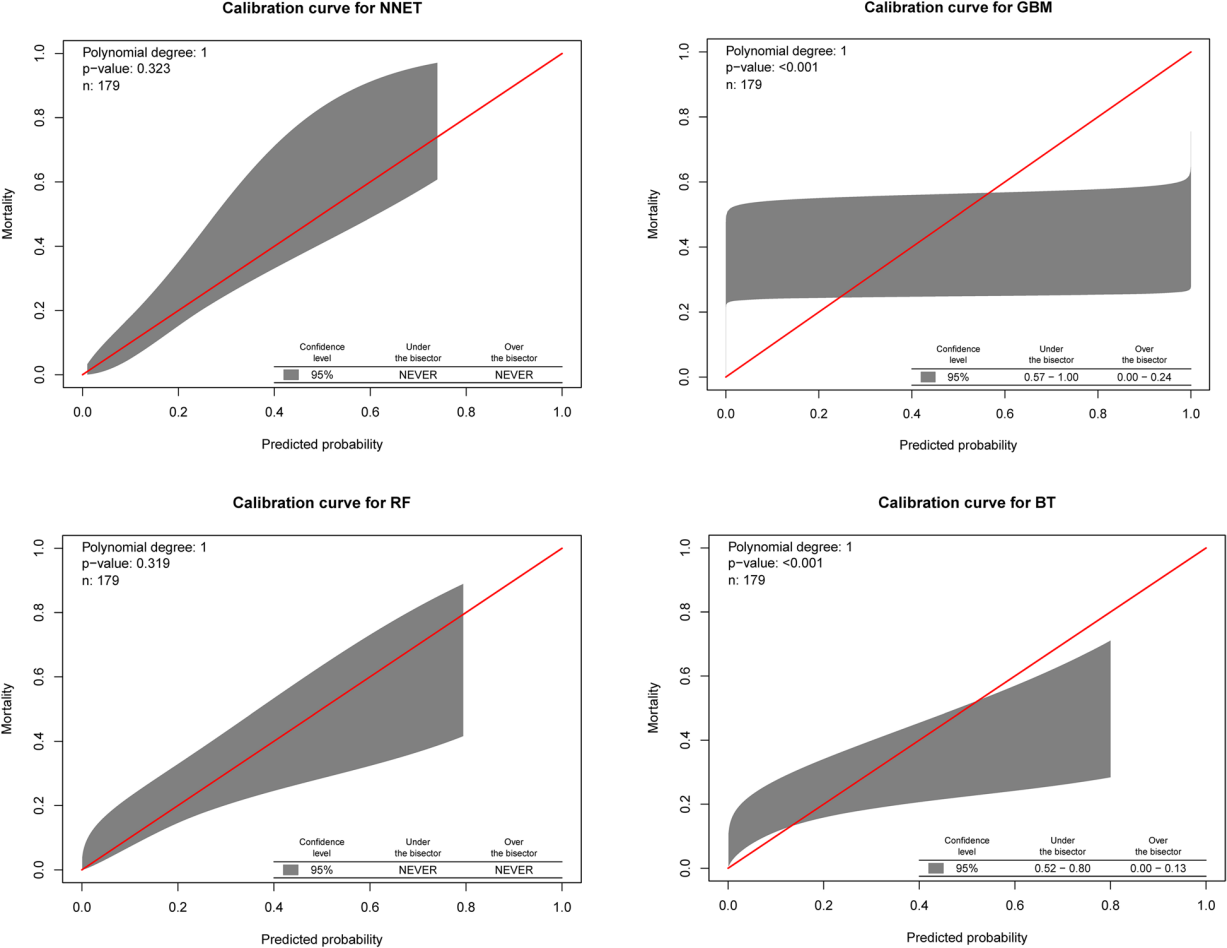
The calibration curve for different machine learning models in the validation cohort. NNET, artificial neural network; GBM, gradient boosting machine; RF, random forest; BT, bagged trees

Figure 3 showed the calibration curve for the calibration performance. The Hosmer–Lemeshow goodness-of-fit test was also calculated. To be specific, the chi-squared value was calculated based on the observed and model-predicted values for each group, and the corresponding *P*-value was subsequently obtained. A good fit of the prediction model was indicated if the 45° diagonal bisector did not cross the 95% CI region whereas a *P*-value < 0.05 for the belt plot of the calibration curve indicated a poor fit of the prediction model. The NNET model had good calibration, with *P*-values of 0.323.

**Fig. 4:**
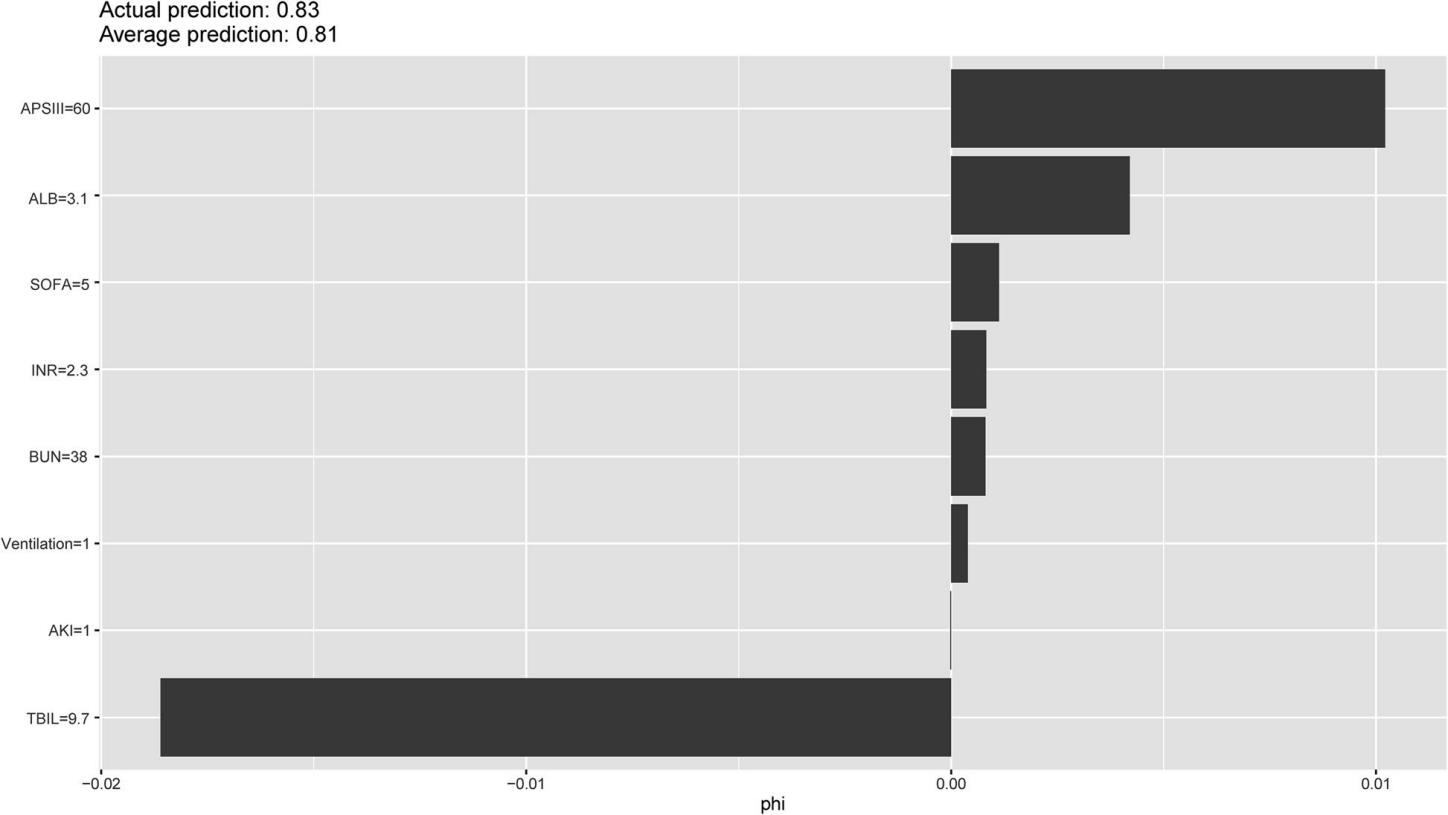
The Shapley values for different variables in the NNET model. APSIII, acute physiology score III; ALB, albumin; SOFA, sepsis related organ failure assessment; INR, international normalized ratio; BUN, blood urea nitrogen; AKI, acute kidney injury; TBIL, total bilirubin; NNET, artificial neural network

**Fig. 5:**
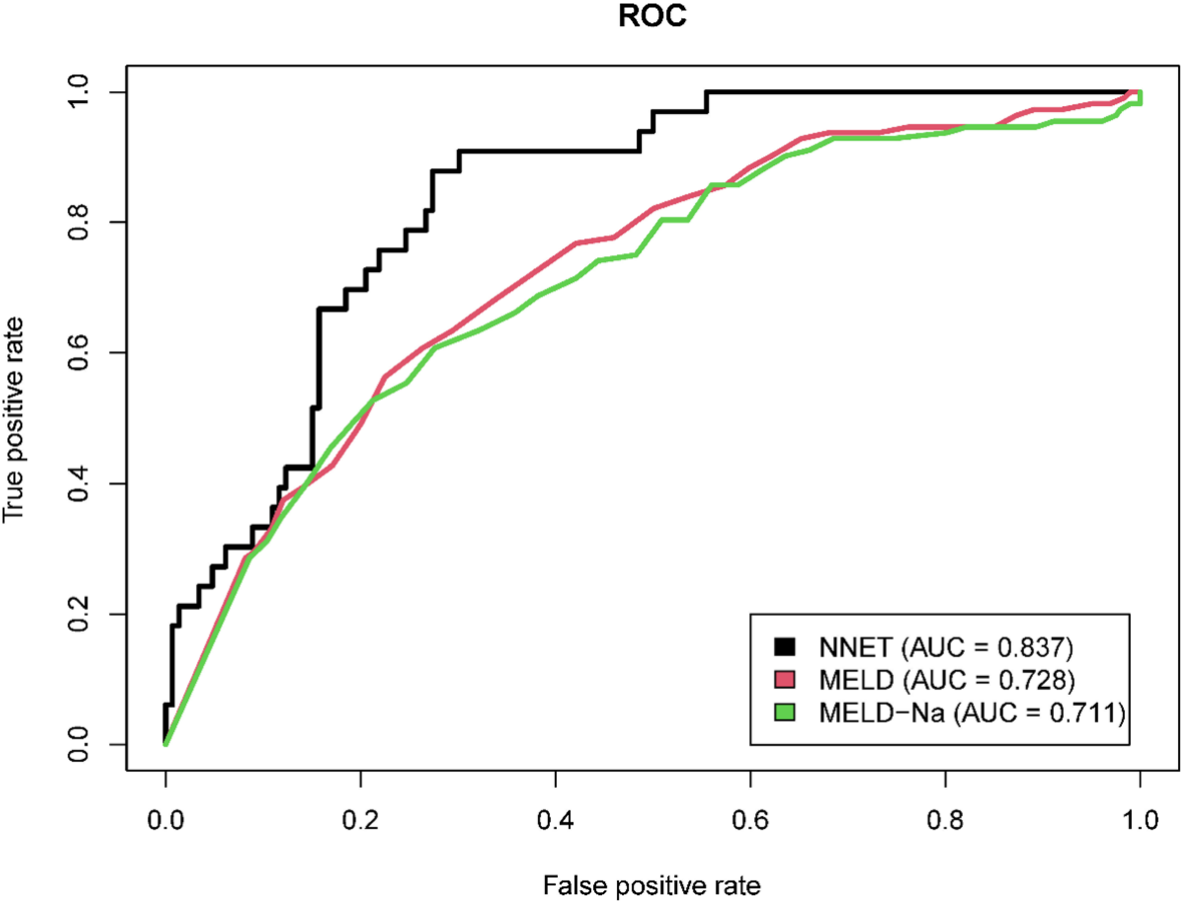
The ROC curves of NNET, MELD and MELD-Na. ROC, receiver operating characteristic; NNET, artificial neural network; MELD, Model for End-Stage Liver Disease; MELD-Na, the sodium for end-stage liver disease

The 8 predictor variables in the NNET model are demonstrated in Fig. 4. Each variable in the study had a different Shapley value for 28-day mortality based on the ML approach. In general, TBIL, APSIII, and albumin were variables with relatively higher Shapely values across the NNET model. More precisely, these variables have a higher impact on the outcome of the model. Additionally, the AUCs of MELD and MELD-Na in the prediction of 28-day death were 0.728 (95% CI: 0.677–0.779) and 0.711 (95% CI: 0.658–0.765), respectively (Fig. 5).

## Discussion

Liver cirrhosis results from the development of various acute and chronic liver diseases, and HE is a common critical complication of decompensated cirrhosis. This retrospective study analyzed a relatively large population of MIMIC-IV, and found 8 variables were independent predictors of 28-day death. Notably, in this study, all indicators were obtained within the first 24 h of ICU admission, providing a short window for identifying severe patients with HE. Additionally, among the four ML models that were validated, NNET was the best model, with good discriminative (AUC = 0.837) and calibration ability simultaneously. Our model could potentially be useful to clinicians in their decision-making when it comes to the selection of therapeutic strategies. Features obtained in the final model included APSIII, SOFA, INR, TBIL, albumin, BUN, AKI and mechanical ventilation, which is consistent with the findings of other published studies.

In a recent research, researchers also identified SOFA and systemic inflammatory response syndrome (SIRS) as factors associated with 30-day mortality in patients with HE [18]. Patients with HE are functionally immuno-suppressed and susceptible to infection [19], which is a frequent precipitant for organ dysfunction. Likewise, the resultant organ failures as indicated by high SOFA and APSIII means significant mortality [20, 21]. Changes in TBIL and albumin often reflect liver function in patients with liver cirrhosis, which is closely associated with a poor prognosis [22]. Peng Y et al. in their study concluded that TBIL was independently correlated with in-hospital death in cirrhotic patients with HE [23]. Bai Z et al. reported that albumin level was an independent risk factor for HE-associated mortality during hospitalizations in cirrhosis (OR = 0.864, 95% CI = 0.771–0.967) [24]. Other studies have also validated this finding [25–28]. By conducting a preliminary observation, Udayakumar N et al. found that high serum bilirubin values in chronic liver disease were simple parameters that would predict a poor outcome in patients with HE [9]. Moreover, serum bilirubin levels as prognostic biochemical markers have been reported elsewhere and are similar to our observations [29, 30]. One previous investigation identified elevated levels of TBIL, BUN, and decreased albumin as factors associated with poor prognosis in patients with acute HE [23]. In addition, Hung TH et al. in their study also identified that AKI increased the 3-year mortality of cirrhotic patients with HE [31]. Other study also found that AKI was the main independent predictor of ICU death and 1-year mortality [32]. Possible mechanisms may be that the increased ammonia from blood stream exacerbates cerebral edema in cirrhotic patients with HE [33–35]. Additionally, according to Cui Y et al., hepatorenal syndrome (HRS) was independent factor associated with 3-month death [36]. Notably, decreased kidney function usually accompanies end-stage liver disease, including HRS. Renal dysfunction can increase BUN, and in turn, resultant diffusion of BUN into the intestine can cause enhanced ammonia uptake accompanied by worsening of HE [36]. Previous studies explored the prognostic factors correlated with 180 cirrhotic patients presenting with HE who were admitted to ICU. And researchers also found that the use of mechanical ventilation was a significant risk factor for mortality [37]. The study by Saffo S et al. has demonstrated that mechanical ventilation was the strongest predictor of in-hospital mortality in their primary analysis (OR, 3.00; 95% CI, 2.14–4.20; *P* < 0.001) and in all sensitivity analyses [38]. Studies by Benhaddouch Z et al. came to similar conclusion [8]. This may be due to the fact that patients with hepatic encephalopathy are at a greater risk for the complications of mechanical ventilation because of possible underlying circulatory, neurologic, and immunologic disturbance [39]. Mechanical ventilation itself may aggravate the existing condition, manifested as impairment in cardiovascular and cognitive function and immune defense, consequently, patients with hepatic encephalopathy may particularly be susceptible to developing such complications as shock, progressive delirium, and infection [40, 41]. Although MELD and MELD-Na are accurate, highly specific scores that are commonly used to reveal liver disease severity and quantify mortality risk [42, 43], the predicative performance for 28-day mortality of these scores in patients with HE remains unclear. Hence, we performed the ROC curves in this study, and found that the AUCs of the MELD and MELD-Na scores for 28-day mortality was lower than that of NNET. which suggested our model was superior to other models in terms of diagnostic discrimination. The reason for this may be, that only simple indicators (creatinine, bilirubin, and INR) cannot precisely assess the degree of cirrhosis [44, 45]. Moreover, a frequently reported drawback of the MELD score was that it has the disadvantage of lacking objective parameters reflecting the patient 's physical and nutritional status, including albumin [46].

There were several strengths in this study. First, multiple ML algorithms were used to build a predictive model. Third, 8 variables selected for the final model are readily available in clinical practice, enabling the model to be implemented easily in the real world.

This study had several limitations. First, because the model was based on MIMIC-IV, which is a single-center database, it still needs to be externally validated in other datasets. Secondly, all the variables were first obtained after an ICU admission, without considering that indicators were dynamically changing. Thirdly, originated from a retrospective cohort, the model needs further prospective validation before being considered for clinical application. Lastly, the majority of the patients included in this study were White, therefore, these findings may not be extrapolated to other populations, such as Asians.

## Conclusions

In this study, we proposed an individualized predictive model based on ML for 28-day mortality in HE patients upon ICU admission. We demonstrated that APSIII, SOFA, mechanical ventilation, INR, TBIL, albumin and AKI are crucial for predicting 28-day mortality. The model in our study had superior performance to MELD score or MELD-Na score predicting 28-day mortality. In the future, real-time prediction of mortality risk among HE patients might be realized, which, in turn, will optimize treatment to improve clinical prognosis.

## Supplementary Information

**Additional file 1: Table S1.** Missing number (%) for included variables in the dataset.

## Data Availability

Publicly available datasets were analyzed in this study. These data can be found at Physionet (https://physionet.org/content/mimiciv/2.0/).
