# Supplementary material for: Using machine learning methods to predict 28-day mortality in patients with hepatic encephalopathy
Source: BMC Gastroenterol. 2023 Apr 6;23:111. doi: 10.1186/s12876-023-02753-z (PMC10077693; doi:10.1186/s12876-023-02753-z)
Supplement: Supplementary file 1 — Additional file 1: Table S1. Missing number (%) for included variables in the dataset. [file 12876_2023_2753_MOESM1_ESM.docx]

| Table S1 Missing number (%) for included variables in the dataset | |
| --- | --- |
| Variables | Missing, N (%) |
| Temperature (°C) | 2.50 |
| MAP (mmHg) | 0.50 |
| Heart rate (min) | 0.50 |
| Respiratory rate (min) | 0.83 |
| RBC (10^9^/L) | 0.17 |
| WBC (×10^9^/L) | 0.17 |
| HGB (g/dl) | 0.17 |
| PLT (×10^9^/L), median | 0.17 |
| RDW (%) | 0.17 |
| HCT (%) | 0.17 |
| APTT (seconds) | 3.00 |
| PT (s) | 2.33 |
| INR | 2.33 |
| Bicarbonate (mmol/L) | 0.17 |
| Lactate (mmol/L) | 27.12 |
| Aniongap | 0.17 |
| Calcium (mmol/L) | 0.83 |
| Glucose (mmol/L) | 0.17 |
| TBIL (μmol/L) | 5.32 |
| Albumin (mmol/L) | 19.47 |
| ALT (U/L) | 5.49 |
| AST (U/L) | 5.16 |
| ALP (U/L) | 5.66 |
| Urine output (ml) | 2.66 |

MAP, mean artery pressure; RBC, red blood cell; WBC, white blood cell; HGB, hemoglobin; PLT, platelet; RDW, red cell distribution width; HCT, hematocrit; APTT, activated partial thromboplastin time; PT, prothrombin time; INR, international normalized ratio; TBIL, total bilirubin; ALT, alanine transaminase; AST, aspartate transaminase; ALP, alkaline phosphatase
